# Re-evaluation of the discriminatory power of DNA barcoding on some specimens of African Cyprinidae (subfamilies Cyprininae and Danioninae)

**DOI:** 10.3897/zookeys.746.13502

**Published:** 2018-03-26

**Authors:** Mariam I. Adeoba, Ronny Kabongo, Herman Van der Bank, Kowiyou Yessoufou

**Affiliations:** 1 Department of Zoology, University of Johannesburg, Kingsway Campus PO Box 524, Auckland Park 2006, South Africa; 2 African Centre for DNA Barcoding, University of Johannesburg, Kingsway Campus, PO Box 524, Auckland Park 2006, South Africa; 3 Department of Geography, Environmental management and Energy studies, University of Johannesburg, Kingsway Campus PO Box 524, Auckland Park 2006, South Africa

**Keywords:** BRONX algorithm, character variations, COI, specimen identification

## Abstract

Specimen identification in the absence of diagnostic morphological characters (e.g., larvae) can be problematic even for experts. The goal of the present study was to assess the performance of COI in discriminating specimens of the fish family Cyprinidae in Africa, and to explore whether COI-phylogeny can be reliably used for phylogenetic comparative analysis. The main objective was to analyse a matrix of COI sequences for 315 specimens from 15 genera of African Cyprinidae using various distance-based identification methods alongside multiple tests of DNA barcode efficacy (barcode gap, species monophyly on NJ tree). Some morphological and biological characters were also mapped on a COI-phylogeny reconstructed using Maximum Parsimony. First, the results indicated the existence of barcode gaps, a discriminatory power of COI ranging from 79 % to 92 %, and that most nodes form well-supported monophyletic clades on an NJ tree. Second, it was found that some morphological and biological characters are clustered on the COI-phylogeny, and this indicates the reliability of these characters for taxonomic discrimination within the family. Put together, our results provide not only an additional support for the COI as a good barcode marker for the African Cyprinidae but it also indicate the utility of COI-based phylogenies for a wide spectrum of ecological questions related to African Cyprinidae.

## Introduction

Cyprinidae is the most diverse family of freshwater fishes (Nelson et al. 2006; Imoto et al. 2013) with 377 genera and over 3000 described species (Eschmeyer and Fong 2015; Froese and Pauly 2017). Species of this family are mostly found in Africa, Europe, Asia, and North America (Thai et al. 2007). In Africa, recent studies of the family have identified 24 genera and 539 species (Yang et al. 2015; Vreven et al. 2016). Some species of the family are of economic importance in aquaculture, angling, fisheries, aquarium trade and many serve as an essential source of protein for humans in addition to their high values in recreational fisheries (Skelton 2001; Thai et al. 2007; Collins et al. 2012).

Traditionally, external morphological and osteological characteristics have been used to differentiate species within the subfamilies Cyprininae and Danioninae (Zhou 1989; Chen et al. 2009; Yang et al. 2010; Liao et al. 2011; Nelson et al. 2016). For example, diagnostic characters such as a spinous anal-fin ray in some Cyprininae (Yang et al. 2010), interhyal not ossified (Liao et al. 2011) and an extended anal fin in mature males of some Danioninae (Stiassny et al. 2006) are used for specimen identification in both subfamilies. Additionally, there are key morphological features that distinguish the males from the females, including a brighter breeding colour, longer fins and presence of the tubercles on the body and head in some African genera (Skelton 2001). Similarly, morphological features such as the presence/absence of barbels, the number of barbels, as well as barbel type, pattern of innervation, and barbel position have been used to differentiate species within and between genera of the subfamilies Cyprininae sensu lato and Danioninae sensu lato (Howes 1991; Skelton 2001).

The taxonomy of the family has been a topic debated in several studies (e.g., Howes 1991; Cavender and Coburn 1992; Briolay et al. 1998; Zardoya and Doadrio 1999; Gilles et al. 2001; Yang et al. 2015; Ren and Mayden 2016). Some studies have explored the phylogeny of this family at subfamily and genus levels using both mitochondrial and nuclear genes (Zardoya and Doadrio 1999; Simons et al. 2003; Stiassny and Getahun 2007; Tang et al. 2010; Yang et al. 2010; Zheng et al. 2012; Yang et al. 2015). Specifically in Africa, most cyprinid species were previously assigned to the subfamily Cyprininae (Tsigenopoulos et al. 2002). The former genus *Barbus* forms a large polyphyletic group of more than 800 species across the world and 300 species across Africa (Skelton et al. 1991). Early studies used, in addition to morphological characteristics, the ploidy level to reorganise the genus *Barbus* sensu lato in Africa (Agnèse et al. 1990; Oellermann and Skelton 1990; Güegan et al. 1995; Berrebi et al. 1996; Machordom and Doadrio 2001; Tsigenopoulos et al. 2002). As a result, some African *Barbus* from northern and southern Africa have been regrouped into genera such as *Luciobarbus* and *Pseudobarbus* (Swartz et al. 2009; Tsigenopoulos et al. 2010) and other species now belong to genus *Labeobarbus* (Oellermann and Skelton 1990; Berrebi et al. 1996; Machordom and Doadrio 2001).

Similarly, the recent molecular and morphological work of Yang et al. (2015) on subfamily Cyprininae had led to a major reclassification and name changes in the global Cyprinidae. This reclassification has since been adopted in some recent works (Armbruster et al. 2016; Decru et al. 2016; Skelton 2016; Vreven et al. 2016). As a result, some genera within the African Cyprinidae are now subfamilies (e.g., Cyprininae, Danioninae and Leuscininae) with few species belonging to non-specified subfamilies (Suppl. material 1). Presently, the African Cyprininae is grouped into four tribes including Barbini, Smiliogastrini, Torini and Labeonini (Yang et al. 2015). The tribe Barbini includes genera such as *Luciobarbus*, *Barbopsis*, *Caecobarbus* and *Coptostomabarbus* and the Smiliogastrini includes the genera *Barbodes*, *Barboides*, *Clypeobarbus*, *Enteromius* and *Pseudobarbus*. The former African diploid ‘*Barbus*’ is now reclassified within the genus *Enteromius* (Yang et al. 2015; Armbruster et al. 2016) and the South African tetraploid *Barbus* has been elevated to genus ‘*Pseudobarbus*’ (Yang et al. 2015; Skelton 2016), although Schmidt and Bart (2015) suggested a revision for genus *Pseudobarbus* to clarify those with inverted comma. Additionally, the former African *Varicorhinus* was reassigned to *Labeobarbus* in the tribe Torini (Beshera et al. 2016; Skelton 2016; Vreven et al. 2016). Yang et al. (2015) also suggested *Sanagia velifera* Holly, 1926 to be grouped with the genus *Labeobarbus*. The tribe Labeonini includes the genera *Labeo*, *Garra* and *Prolabeo* (Rainboth et al. 2012; Yang et al. 2012, 2015). In such context of taxonomic debate around the family Cyprinidae (Yang et al. 2015), it becomes necessary to question whether the ongoing global campaign of DNA barcoding can play a role at least in assigning specimen to their corresponding taxa. The DNA barcoding approach has been employed to complement or refine morphological species identification (Kochzius et al. 2010; Pereira et al. 2011; Chen et al. 2015). DNA barcoding is based on the use of a short standardised cytochrome c oxidase subunit I (COI) sequence to distinguish between animal species (Hebert et al. 2003; 2004). It has gained worldwide support because it is rapid, cost-effective (but see Stein et al. 2014), and applicable to species identification across the animal kingdom (e.g., Hebert et al. 2003; Ward et al. 2005; Van der Bank et al. 2013; Sethusa et al. 2014; Decru et al. 2016; Nigro et al. 2016). In particular, Decru et al. (2016) clearly demonstrated, using DNA barcoding, how knowledge of the African fish species diversity can be greatly improved, but they focused only on the Congo Basin region in Central Africa.

The present study uses a broader sampling of the African Cyprinidae and integrates morphology and ploidy data to further assess the effectiveness of DNA barcoding in discriminating specimens within the family. Specifically, the aim was to: (i) test the reliability of COI as a DNA barcode for the African Cyprinidae based on barcode gap, various distance methods, and the Rosenberg test of species monophyly; and (ii) map six traits including five morphological characters and ploidy level onto a COI-based phylogeny of the African Cyprinidae.

## Materials and methods

### Sample collections

First, 584 COI sequences of the African Cyprinidae specimens were retrieved from the Barcode of Life Database (BOLD; www.boldsystems.org) and GenBank/EBI (www.ncbi.nlm.nih.gov/nuccore). Some of the sequences from BOLD had been generated from our group (African Centre for DNA Barcoding) (Suppl. material 1). Second, for the purpose of the present study, an additional set of 55 new sequences of southern African specimens were generated to create a total DNA matrix of 639 specimens consisting of 15 of the 24 genera of African Cyprinidae. Sequences of the 55 specimens are made available on BOLD and GenBank/EBI. The BOLD identification numbers, voucher information, GenBank accession numbers, and species authorities for all species analysed in this study are presented in Suppl. material 2. Localities, images and additional information are also available on BOLD. It should also be noted that, as a result of the ongoing taxonomic revision and debates around this family, some of the African species names have been altered in FishBase but are yet to be updated on BOLD and GenBank. Therefore, for this study the old names were retained in our analysis (see Suppl. material 2; but new names are adopted in Figures 4 and 5). All the species analysed in the present study are those that have accession numbers in Suppl. material 1.

### DNA extraction, amplification, and sequencing of COI

The 55 new COI sequences mentioned above were generated following the manufacturers’ recommended protocol developed from NucleoSpin® Tissue kit (Macherey- Nagel). The sequence amplification (PCR) was done in accord with Hajibabaei et al. (2005). Specifically, PCR reactions were done in a total volume of 25 μL. The master mix consisted of 12.5 μL of top taq, 0.8 μL of BSA, 0.3 μL of both primers and 10.1 μL of dH2O. The DNA templates prepared for the PCR amplification ranged from 1–3 μL, depending on the strength and quality of DNA products visualized from the agarose gel. The PCR conditions were as follows: initial melting for 2 mins at 95 oC, denaturation at 94 oC for 0.5 min, annealing at 52 oC for 0.5 min, extension at 72 oC for 1 min followed by a final extension at 72 oC for 10 mins (35 cycles) and a hold at 4 oC (Steinke and Hanner 2011). The primer pair used was COI-Fish. F1 5’-TCAACCAACCACAAAGACATTGGCAC-3’ and COI-Fish.R1 3’-TAGAC TCTG GGTGGCCAAAGAATCA-5’.

After the amplification, PCR products were visualised on 1.5% agarose gels. Visible products were cleaned using silica column kits, viewed again on agarose gels, and selected for cycle sequencing. Sequencing of COI region was done following the standard protocols of the Canadian Centre for DNA Barcoding (CCDB). Sequences were aligned using Multiple Sequences Comparison by Log-Expectation (MUSCLE vs. 3.8.31; Edgar 2004) and exported as a NEXUS file.

### DNA barcoding analysis

Because some DNA sequences available on public repositories are not reliable (Nilsson et al. 2006), we first used the BRONX algorithm (Barcode Recognition Obtained with Nucleotide eXpose´s; Little 2011) to reanalyse all sequences retrieved from BOLD and GenBank/EBI to refine the dataset prior to our DNA barcoding analysis. Based on the BRONX analysis, we removed from our dataset (of 639 sequences) sequences that are questionable, for a number of reasons, including shared haplotypes between species, shorter sequences, and incomplete identification, etc. Also, species with no duplicates (singletons) were excluded, and as a result, the total samples included in our DNA barcoding analysis comprise 315 sequences for 86 species representing 14 out of the 24 (58 %) recognised genera in Africa (Suppl. material 2).

All barcoding analysis was conducted in the R package SPIDER (species identity and evolution in R) vs. 1.1-1 (Brown et al. 2012) following three criteria: barcoding gap, discriminatory power, and tree based analysis for species monophyly. Two techniques were used in evaluating the “DNA barcode gap” (Meyer and Paulay 2005). Firstly, the mean, median, and range of intraspecific genetic distances were compared against interspecific genetic distance (Meier et al. 2008). Secondly, the approach of Meier et al. (2006) was used to assess barcode gap. This involves matching the lowest interspecific distance against the highest intraspecific distance. Genetic distances were calculated using the Kimura 2-parameter (K2P) model (Kimura 1980).

The discriminatory power of the COI gene was tested with three methods: Best Close Match, Near Neighbour and the BOLD identification (threshID) (Meier et al. 2006; Collins et al. 2012). A good barcode should exhibit a high rate of correct species identification. Prior to the analysis, the optimised threshold for specimen identification was first determined using the R function *localMinima* (Brown et al. 2012) and then applied in the Best Close Match and Near Neighbour identification. The identification success of the traditional 1% threshold of BOLD was additionally tested in comparison to bestCloseMatch (Brown et al. 2012).

To test for species monophyly, a tree based analysis using Rosenberg’s (2007) probability of reciprocal monophyly and a Neighbour-Joining (NJ) phylogram was constructed (Rosenberg 2007). For this purpose, our default was set to be false for singletons and our tree rooted on the longest branch with labels corresponding to species vector (Brown et al. 2012).

### Phylogenetic reconstruction and character mapping

A DNA matrix of 315 COI aligned sequences and three outgroups (Suppl. material 3) was formed, and this matrix used to assemble a phylogeny based on Maximum Parsimony (MP) using PAUP* v4.0b 10 (Swofford 2002) with heuristic searches and 1,000 random-addition sequence replicates and tree-bisection-reconnection branch swapping. The following outgroups were chosen from similar past studies: *Moxostoma breviceps* (Cope, 1870) (De Graaf et al. 2007), *Pseudorasbora parva* (Temminck & Schlegel, 1846) and *Gyrinocheilus aymonieri* (Tirant, 1883) (He et al. 2008) (Suppl. material 3).

Information related to morphological characters and ploidy levels were collected from several sources and presented in Suppl. material 3. We selected six characters based on previous studies: number of anal and dorsal fin rays, number of barbels, presence or absence of barbels, length, ploidy levels, and type of lips (Howes 1991, Skelton 2001; Zheng et al. 2010; Yang et al. 2015). Character states were tabulated and mapped using Mesquite 3.04 (Maddison and Maddison 2015) onto the parsimonious molecular phylogenetic tree.

## Results

**Figure 1.**
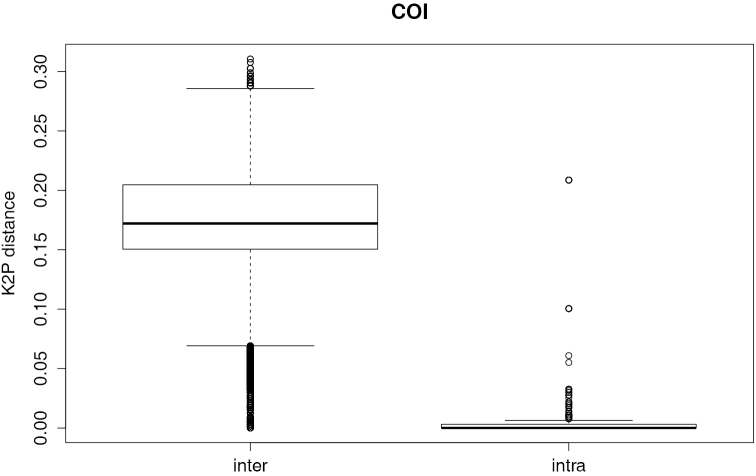
Evaluation of barcode gap in the dataset. Boxplot of the interspecific (inter) and intraspecific genetic (intra) distances, indicating the existence of a barcode gap, i.e., interspecific distance is larger than intraspecific distance. The median is indicated by the horizontal line and the range as the vertical dashed lines and outliers by bold vertical lines.

**Figure 2.**
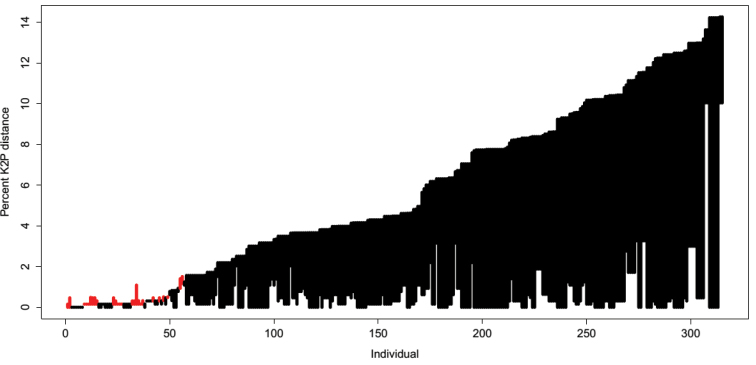
Evaluation of barcode gap in the dataset. Line plot of the barcode gap for the 315 Cyprinidae individuals. The black lines indicate where the smallest interspecific distance is longer than the longest intraspecific distance (bottom of line value), thus showing the existence of a barcode gap. The red lines show where this pattern is reversed.

**Figure 3.**
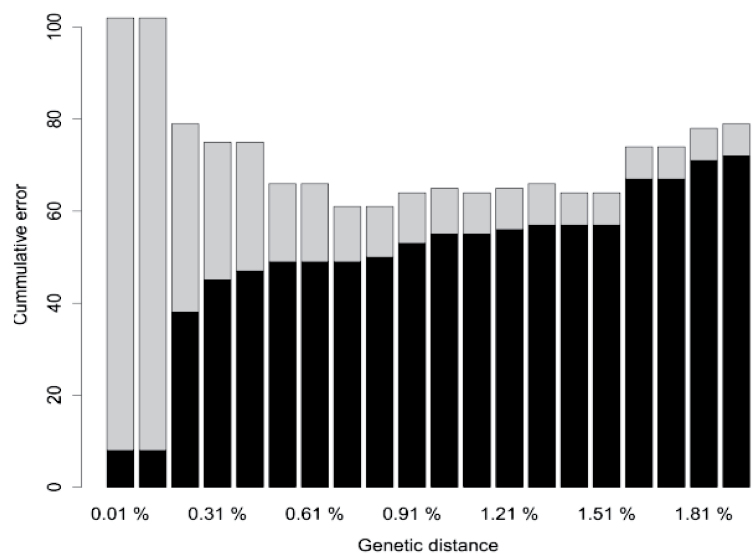
Determination of the threshold genetic distance for species identification. False positive (grey) and false negative (black) identification error rates summed across a range of distance thresholds from 0.01 to 1.9 %. The cumulative error plot indicates the transition between intraspecific and interspecific distances, the genetic distance corresponding to the least cumulative error (1.51 %) showing the appropriate threshold value for the dataset.

The length of the aligned COI matrix was 652 bp with the following base composition: A: 25.9 %, C: 26.8 %, G: 18.2 % and T: 29.1 %. The interspecific genetic distances (K2P) ranged from 0 to 0.30 (median = 0.15) and are larger than the intraspecific genetic distances (range: 0 – 0.02; median = 0.001; p < 0.001; Figure 1). This is indicative of a barcode gap in the COI dataset of the studied Cyprinidae. The existence of a barcode gap is further confirmed when we compared the lowest interspecific versus the furthest intraspecific distance (Figure 2). We found the optimised distance d = 0.015 suitable for species discrimination in the studied African Cyprinidae (Figure 3). Based on this threshold, the performance of COI varies with the method used (Table 1). The near neighbour method shows a discriminatory power of 92.1 %. The other two methods provide a lower performance of 88.2 % for the best close match (278 specimens out of 315) and 79.4 % with the BOLD method.

**Figure 4.**
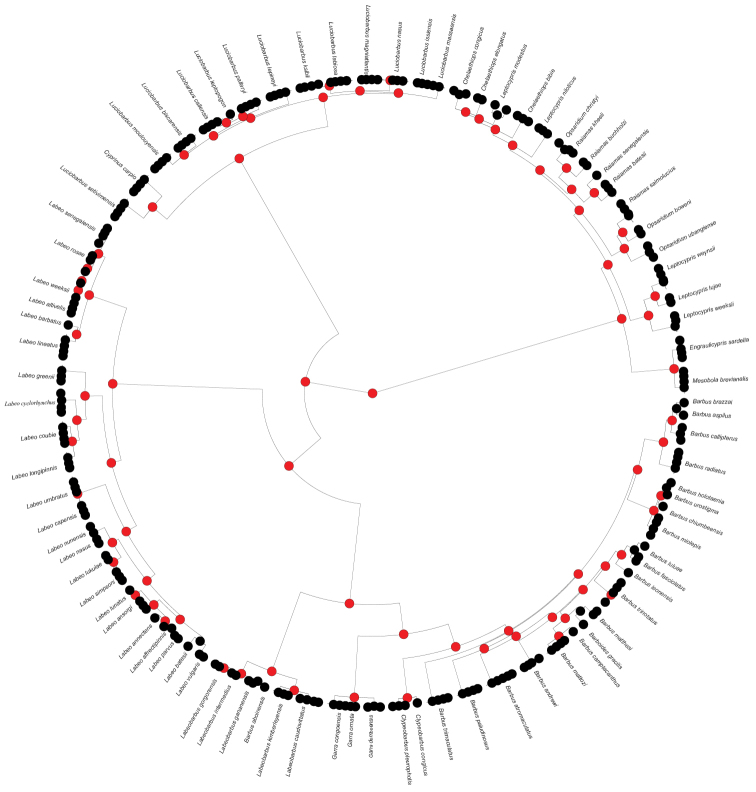
Neighbour-joining tree analysis using Rosenberg’s (2007) test. Nodes in red are strongly supported nodes, indicating species monophyly.

**Figure 5.**
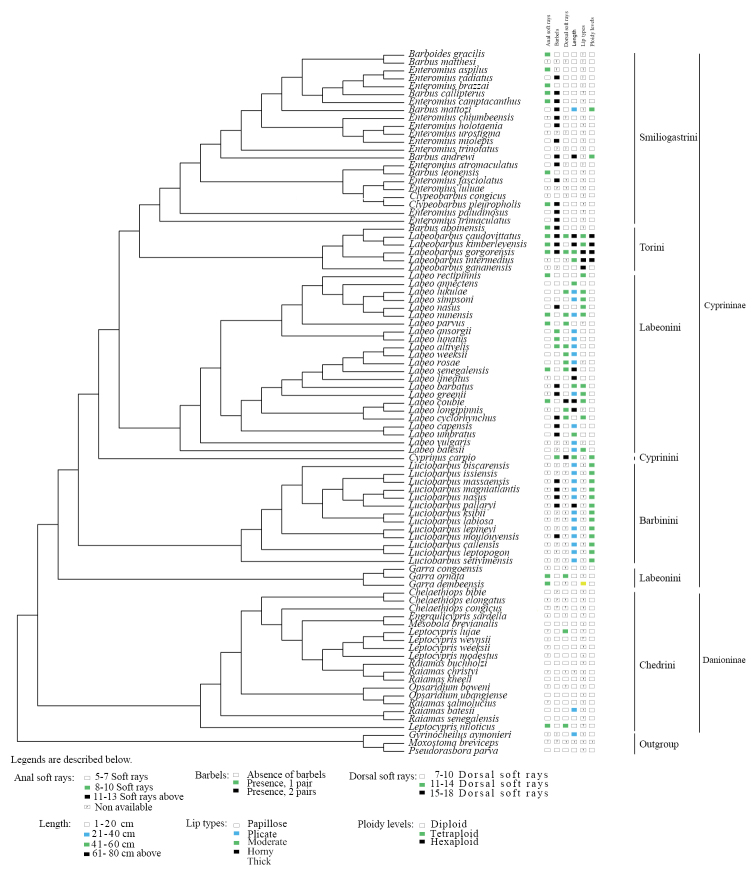
Parsimonious phylogenetic tree showing some plotted morphological characters, such as anal soft rays, barbels, dorsal soft rays, length, lip types, and ploidy status of 86 African Cyprinidae species.

In addition, the result presented in Figure 4 shows that most nodes form robust monophyletic clades (red-coded nodes in Figure 4). The level of monophyly is further confirmed on Figure 5 which clearly indicates two distinct subfamilies (Cyprininae and Danioninae) and five tribes in the subfamily Cyprininae (Figure 5). The mapping of morphological characters and ploidy level on the phylogeny indicates that some characters are clearly clustered [e.g., number of anal soft rays and presence/absence of barbels for the tribe Smiliogastrini, the fish length (21–40 cm) for the tribe Labeonini and the tetraploidy for Barbinini; fig. 5].

**Table 1.** Tests of barcoding identification accuracy with numbers (N) and percentages (%) of each score Near neighbour, Best Close Match and Bold criteria.

Methods	Near neighbour		Best Close match				Bold criteria			
Scores	False	True	Ambiguous	Correct	Incorrect	No ID	Ambiguous	Correct	Incorrect	No ID
N	25	290	10	278	17	10	57	250	1	7
(%)	7.9	92.1	3.2	88.2	5.4	3.2	18.1	79.4	0.3	2.2

## Discussion

Although COI is a universally accepted DNA barcode for animal groups (Hebert et al. 2003), its efficacy has also been questioned for some clades (Vences et al. 2005a; Chen et al. 2012; Murphy et al. 2013), and this prompts the need to assess its reliability for any particular group of interest (Collins et al. 2012).

The results presented in this work confirm that COI can be reliably used from a barcode perspective to distinguish between specimens of the African Cyprinidae in a dataset of 315 specimens representing 14 out of the 24 (58 %) recognised genera in Africa. For example, a significant barcode gap was found irrespective of the methods used, and this has also been reported for Cyprinidae of other geographic regions (e.g., Batishchevaa et al. 2011). Our results (79.4 %–92.1 %) from the distance-based method showed a pattern similar to the 90 % to 99 % discriminatory power reported for ornamental cyprinid fish species also mostly from Cyprininae and Danioninae and a catostomid (Collins et al. 2012). Irrespective of some drawbacks associated with the use of DNA barcoding and highlighted by some authors for some taxonomic groups (Vences et al. 2005a, b; Nielsen and Matz 2006; Valentini et al. 2008; Laskar et al. 2013) as well as the recent development of new generation sequencing techniques (e.g., Taylor and Harris 2012), the marker COI still remains useful for identification purposes (Batishchevaa et al. 2011; Collins et al. 2012; Van der Bank et al. 2013).

For example, the high level of COI discrimination is further supported by the test of species monophyly, a test that resulted in strongly supported clades based on Rosenberg (2007)’s probability of reciprocal monophyly on the NJ tree (see also Collins et al. 2012). Even our Maximum Parsimony tree provides additional support to the COI’s power of discriminating between clades of the African Cyprinidae. Specifically, our phylogenetic analysis retrieved 14 monophyletic genera clearly grouped into two subfamilies (Cyprininae and Danioninae). Within the Cyprininae, five tribes are distinctly recovered: Barbini, Cyprinini, Labeonini, Smiliogastrini, and Torini as in Yang et al. (2015). The subfamily Danioninae was represented in our material by the tribe Chedrini which is well supported and includes *Chelaethiops*, *Engraulicyprus*, *Leptocypris*, *Mesobola*, *Opsaridium*, and *Raiamas* (see also Tang et al. 2010).

This evidence of monophyly accords with the morphology-based taxa delimitation as we found that some morphological characters and ploidy levels clustered within some clades along the phylogeny. Such characters that clustered within clades include, for example, the number of anal soft rays and presence/absence of barbels for the tribe Smiliogastrini, the fish length (21–40 cm) for the tribe Labeonini and the tetraploidy for Barbinini. Such clustering on the COI-phylogeny is evidence not only for COI as DNA barcoding of some African Cyprinidae 11 a good barcode for the family Cyprinidae but also that COI-phylogeny can be used for a comparative phylogenetic analysis. Only the tribe Labeonini sensu Rainboth 1991 (Yang and Mayden 2010) was retrieved non-monophyletic in our dataset.

Overall, the existence of DNA barcode gap and a high discriminatory power, as well as the high level of monophyly give support to the use of COI as a reliable DNA barcode for African Cyprininae and Danioninae. Several studies have examined the phylogeny of this family at subfamily and genus levels using both mitochondrial and nuclear genes (Simons et al. 2003; Stiassny and Getahun 2007; Swartz et al. 2009; Tsigenopoulos et al. 2010; Yang et al. 2010; Zheng et al. 2012). Our study provides additional evidence for the effectiveness of DNA barcode data as a complementary tool to morphology-based identification of some African Cyprinidae, and our findings indicate that a COI-based phylogenetic tree for the African Cyprinidae can be used in comparative phylogenetic analyses and important applied problems (e.g., conservation) for this group of fish.

## Supplementary materials

Supplementary material 1 Table S1 Data type: occurence Explanation note: Taxonomy of African Cyprinidae and geographic origins. This is an open access article distributed under the terms of the Creative Commons Attribution License (CC BY 4.0), which permits unrestricted use, distribution, and reproduction in any medium, provided the original author and source are credited.Adeoba MI, Kabongo R, Van der Bank H, Yessoufou K

Supplementary material 2 Table S2 Data type: occurence Explanation note: List of 315 Cyprinidae specimens analysed in this study. Full names, voucher information, and geographic origins are presented. Accession numbers for ACDB (African Centre for DNA Barcoding at the University of Johannesburg), GenBank, and BOLD are also included. This is an open access article distributed under the terms of the Creative Commons Attribution License (CC BY 4.0), which permits unrestricted use, distribution, and reproduction in any medium, provided the original author and source are credited.Adeoba MI, Kabongo R, Van der Bank H, Yessoufou K

Supplementary material 3 Table S3 Data type: measurement Explanation note: Matrix of the morphological characters used for character mapping along a phylogeny. Details of the characters (1–6) and sources are provided at the bottom of the table.. This is an open access article distributed under the terms of the Creative Commons Attribution License (CC BY 4.0), which permits unrestricted use, distribution, and reproduction in any medium, provided the original author and source are credited.Adeoba MI, Kabongo R, Van der Bank H, Yessoufou K
