# Supplementary material for: Re-evaluation of the discriminatory power of DNA barcoding on some specimens of African Cyprinidae (subfamilies Cyprininae and Danioninae)
Source: Zookeys. 2018 Mar 26;(746):105–21. doi: 10.3897/zookeys.746.13502 (PMC5906743; doi:10.3897/zookeys.746.13502)
Supplement: Supplementary material 2 — Table S2 [file zookeys-746-105-s002.docx]

**Table S2**: List of 315 Cyprinidae specimens analyzed in this study. Full names, voucher information and geographic origin are presented. Accession numbers for ACDB (African Centre for DNA Barcoding at the University of Johannesburg), GenBank and BOLD are also included.

| Subfamilies | Genera | Taxa and designated authority | Process Identity or Accession no | Origin of sequence | Geographic origin |
| --- | --- | --- | --- | --- | --- |
|  | *Barboides* | *Barboides gracilis* ([Brüning](http://en.wikipedia.org/w/index.php?title=Christian_Br%C3%BCning&action=edit&redlink=1" \o "Christian Brüning (page does not exist)), 1929) | RCYY457-11  RCYY458-11 | BOLD | Benin |
|  | *Chelaethiops* | *Chelaethiops bibie* (Joannis, 1835) | AMNHI253-10  ANGBF2688-12  HM224141.1  HM418210.1 | BOLD  BOLD  GenBank  GenBank | Nile River and Webi Shebeli. Also present in the Niger, Bénoué and Volta Basin. |
|  |  | *Chelaethiops congicus* (Nichols & Griscom, 1917) | AMNHI190-10  AMNHI191-10  AMNHI291-10  AMNHI292-10 | BOLD | Congo River System, from the Lower Congo up to Lufira and Lake Mweru and in Lake Tanganyika |
|  |  | *Chelaethiops elongatus* [Boulenger](http://en.wikipedia.org/wiki/George_Albert_Boulenger), [1899](http://researcharchive.calacademy.org/research/ichthyology/catalog/getref.asp?id=589) | AMNHI037-09  AMNHI065-09  ANGBF2695-12 | BOLD | Congo River System, from the Lower Congo up to the upper Lualaba, in Democratic Republic of the Congo, Republic of Congo and Central African Republic |
|  | *Clypeobarbus* | *Clypeobarbus congicus* [Boulenger](http://en.wikipedia.org/wiki/George_Albert_Boulenger), [1899](http://researcharchive.calacademy.org/research/ichthyology/catalog/getref.asp?id=589) | AMNHI082-09  KP712041.1 | BOLD  GenBank | Congo River Basin from the Lower Congo at Matadi up to the Upper Lualaba, in Democratic Republic of the Congo |
|  |  | *Clypeobarbus pleuropholis*  [Boulenger](http://en.wikipedia.org/wiki/George_Albert_Boulenger), [1899](http://researcharchive.calacademy.org/research/ichthyology/catalog/getref.asp?id=589) | DCF284-15  DCF747-15  KT193024.1  KT193487.1 | BOLD  BOLD  GenBank  GenBank | Chad Basin and Congo Basin, also known from Lower Congo to Upper Lualaba |
|  | *Cyprinus* | *Cyprinus carpio* Linnaeus, 1758 | HVDB257-10  HVDB296-10  HVDB297-10  HVDB298-10  HVDB304-10 | SAIAB | Wide spread throughout southern Africa, but absent from mountain areas and restricted in warmer tropical areas such as the Lowveld. |
|  | *Engraulicypris* | *Engraulicypris sardella* (Günther, 1868) | AMNHI229-10  AMNHI230-10  ANGBF2687-12  HM418189.1  HM418190.1 | BOLD  BOLD  BOLD  GenBank  GenBank | Occurs in Lake Malawi, Upper Shire and Lake Nyasa |
|  | *Enteromius* | *Enteromius aboinensis* [Boulenger](http://en.wikipedia.org/wiki/George_Albert_Boulenger), 1911 | AMNHI156-10  AMNHI158-10 | BOLD | Nigeria and Cameroon |
|  |  | *Enteromius aspilus* Boulenger, 1907 | GBGCA6615-15  KP71203-41 | GenBank | Rivers Dja (Middle Congo River Basin), Nyong, and Sanaga in Cameroon |
|  |  | *Enteromius atromaculatus* Nichols & Griscom, 1917 | DCF120-15  DCF121-15  DCF182-15  DCF183-15  DCF18415 DCF005-15 | BOLD | Middle Congo River Basin (Lower Congo) in the Drainages of the Ituri, Uele, Itimbiri and Dja, and at Kisangani. |
|  |  | *Enteromius brazzai* Pellegrin, 1901 | DCF297-15  DCF298-15 | BOLD | Central Congo River Basin and from just above the Wagenia Falls (Stanley Falls). |
|  |  | *Enteromius callipterus* Boulenger, 1907 | AMNHI151-10  AMNHI152-10  RCYY446-11  KP712129.1 | BOLD  GenBank | Benin, Burkina Faso, Cameroon, Chad, Congo, Côte d'Ivoire, Gabon, Ghana, Mali, Nigeria, Senegal and Togo |
|  |  | *Enteromius camptacanthus* (Bleeker, 1863) | AMNHI150-10  KP712130.1 | BOLD  GenBank | Widespread from Cameroon to the Chiloango River Basin. Extends westwards as far as the Niger Delta. Also in the Dja Drainage (Middle Congo River Basin) |
|  |  | *Enteromius chiumbeensis* Pellegrin 1936 | AMNHI150-10  HM880206.1 | BOLD  GenBank | Democratic Republic of Congo, Kinshasa |
|  |  | *Enteromius fasciolatus* Günther, 1868 | ANGBF4277-12  RCYY027-11  RCYY028-11 | BOLD | Democratic Republic of Congo, Kinshasa |
|  |  | *Enteromius holotaenia* Boulenger, 1904 | AMNHI313-10  GBGCA7650-15 | BOLD | Democratic Republic of Congo, Kinshasa |
|  |  | *Enteromius leonensis* Boulenger, 1915 | GBGCA4195-13  JX074163.1 | BOLD  Genbank | Present in large Sudanese Basins (Chad, Niger, Volta, Senegal, Gambia), but also in the Comoé and Bandama Rivers in Côte d'Ivoire and Rokel, Jong and Moa Rivers in Sierra Leone |
|  |  | *Enteromius luluae* [Fowler](http://en.wikipedia.org/wiki/Henry_Weed_Fowler), [1930](http://researcharchive.calacademy.org/research/ichthyology/catalog/getref.asp?id=1406) | AMNHI159-10  HM418130.1 | BOLD  Genbank | Lulua River (Kasai Drainage, middle Congo River Basin), Upper Lualaba and Luvua (Upper Congo River Basin) in Democratic Republic of the Congo |
|  |  | *Enteromius matthesi* Poll & Gosse, 1963 | AMNHI195-10  AMNHI196-10  AMNHI197-10 | BOLD | Democratic Republic of Congo, Kinshasa |
|  |  | *Enteromius mattozi* Guimaraes, 1884 | HVDB322-10  HVDB323-10  HVDB324-10  HVDB325-10  HVDB326-10 | (SAIAB) | Limpopo System, Headwaters of Gwai-Zambesi, Kwando-upper Zambezi and Cunene, South Africa |
|  |  | *Enteromius miolepis* Boulenger, 1903 | DCF058-15  DCF065-15  DCF066-15  DCF069-15  DCF070-15 | BOLD | Democratic Republic of Congo, Kasai-Oriental & Kinshasa |
|  |  | *Enteromius paludinosus* Peters, 1852 | HVDBF071-10  HVDBF072-10  HVDBF073-10  HVDBF074-10  HVDBF095-10 | ACDB, UJ | Widespread in east coastal Rivers from East Africa south to the Vungu, KwaZulu-Natal and from southern Congo tributaries and the Quanza in Angola to the Orange |
|  |  | *Enteromius radiatus* Peters, 1853 | HVDBF684-12  HVDBM940-12  HVDBM941-12  HVDBM943-12  HVDBM944-12 | ACDB, UJ | Widespread southwards from Uganda, including Zambian Congo, Cunene, Okavango, Zambezi and east coast Rivers south to the Phongolo System |
|  |  | *Enteromius trimaculatus* Peters, 1852 | HVDBM920-12  HVDBM932-12  HVDBM937-12  HVDBM938-12  HVDBM939-12 | ACDB, UJ | East caost from Ruvuma, Tanzania, to Umvoti in KwaZuluNatal, also Orange, Cunene and Zambian Systems |
|  |  | *Enteromius trinotatus* Fowler, 1936 | DCF112-15  DCF124-15  DCF125-15  KT192852.1  KT192864.1  KT192865.1 | BOLD  BOLD  BOLD  GenBank  GenBank  GenBank | Aruwimi River Drainage in Democratic Republic of the Congo |
|  |  | *Enteromius urostigma* Boulenger, 1917 | KT193462.1  KT193463.1 | GenBank  GenBank | Tributaries of Lake Tanganyika and in the Upper Lualaba |
|  | *Garra* | *Garra congoensis* [Poll](http://en.wikipedia.org/wiki/Max_Poll), [1959](http://researcharchive.calacademy.org/research/ichthyology/catalog/getref.asp?id=3522) | AMNHI201-10  AMNHI202-10  AMNHI203-10 | BOLD | Lower Congo River in Democratic Republic of the Congo |
|  |  | *Garra dembeensis* (Rüppell, 1835) | DCF079-15  DCF080-15  DCF263-15  DCF264-15 | BOLD | Widespread with presence in East Africa, including Ethiopia, Egypt, Kenya and Tanzania, as well as in Cameroon, Nigeria and Chad |
|  |  | *Garra ornata* (Nichols & Griscom, 1917) | AMNHI198-10  AMNHI199-10 GBGCA4156-13 | BOLD | Niger River to Congo River System |
|  | *Labeo* | *Labeo altivelis* [Peters](http://sv.wikipedia.org/wiki/Wilhelm_Peters), 1852 | DCF544-15  DCF705-15  DCF739-15  DCF740-15 | BOLD | Lower and the Middle reaches of the Zambezi River System, including the Shire and Lake Malawi. Also in Lake Mweru and the Luapula (Upper Congo River Basin). Range thought to extend to some East Coast Rivers (Rufiji System) |
|  |  | *Labeo annectens* [Boulenger](http://en.wikipedia.org/wiki/George_Albert_Boulenger), 1903 | AMNHI127-10  HM4181041 | BOLD  GenBank | Principal coastal Rivers of southern Cameroon to Cabinda, and from the Congo River Basin |
|  |  | *Labeo ansorgi* [Boulenger](http://en.wikipedia.org/wiki/George_Albert_Boulenger), 1907 | Kun026  Kun027  Kun028  HVDBF24511 | ACDB, UJ | Cunene and Quanza Systems; Angola and Namibia |
|  |  | *Labeo barbatus* [Boulenger](http://en.wikipedia.org/wiki/George_Albert_Boulenger), 1893 | HM418110.1  HM418111.1 | GenBank | Lower and middle Congo River Basin in Democratic Republic of the Congo and Central African Republic |
|  |  | *Labeo batesii* [Boulenger](http://en.wikipedia.org/wiki/George_Albert_Boulenger), 1911 | GBGC3894-76  GBGC3943-07 | BOLD | Lower Guinea endemic, known from many Rivers of this ichthyogeographical region: Kribi, Mungo, Thsela, Shiloango, Bongola, Ntem, Bitande, Ohumbe, Okano, Mouanda, Kelle, Ogowe, Messok-Messok, Mekay, Lolo and Mvi. |
|  |  | *Labeo capensis* (Smith, 1841) | HVDBF732-12  HVDBF733-12  HVDBF737-12  HVDBF740-12 | ACDB, UJ | Orange-Vaal River System |
|  |  | *Labeo coubie* [Rüppell](http://en.wikipedia.org/wiki/Eduard_R%C3%BCppell), 1832 | BAFEN159-10  BAFEN165-10  BAFEN166-10  BAFEN174-10  BAFEN175-10 | BOLD | Very widespread, within the Drainage Basin of the Nile River, Chad, Niger-Bénoué, Volta, Senegal and Gambia Basins, as well as Cross River and Cameroon coastal Rivers. Also presence in Warri (Nigeria) and Kingani (Tanzania) |
|  |  | *Labeo cyclorhynchus* [Boulenger](http://en.wikipedia.org/wiki/George_Albert_Boulenger), 1899 | AMNHI213-10  AMNHI214-10  DCF342-15  DCF343-15  DCF517-15 | BOLD | Lower and Middle Congo River Basin in Democratic Republic of the Congo and Central African Republic, and in the Ogowe River in Gabon |
|  |  | *Labeo greenii* [Boulenger](http://en.wikipedia.org/wiki/George_Albert_Boulenger), [1902](http://researcharchive.calacademy.org/research/ichthyology/catalog/getref.asp?id=562) | DCF061-15  DCF062-15  DCF207-15  DCF232-15 | BOLD | Congo River Basin, from the lower Congo River up to the Lualaba, in Democratic Republic of the Congo, Central African Republic and Angola |
|  |  | *Labeo lineatus* [Boulenger](http://en.wikipedia.org/wiki/George_Albert_Boulenger), 1898 | DCF510-15  DCF706-15  DCF712-15  DCF741-15  DCF757-15 | BOLD | Congo River Basin, Luapula and Lake Tanganyika and larger Rivers |
|  |  | *Labeo longipinnis* [Boulenger](http://en.wikipedia.org/wiki/George_Albert_Boulenger), 1898 | AMNHI423-11  DCF537-15  DCF550-15  DCF742-15 | ACDB, UJ | Congo River Basin and a single record from Lake Tanganyika |
|  |  | *Labeo lukulae* [Boulenger](http://en.wikipedia.org/wiki/George_Albert_Boulenger), 1902 | DCF037-15  DCF107-15  DCF190-15 | BOLD | Principal coastal Basins of Cameroon to the Chiloango in Democratic Republic of the Congo. In the Congo Basin known from the Kasai and Kwango in Angola, and the Aruwimi and Wagenia Falls in Democratic Republic of the Congo |
|  |  | *Labeo lunatus* Jubb, 1963 | HVDBF038-10  HVDBF043-09 | ACDB, UJ | Upper Zambezi and Okavango Rivers |
|  |  | *Labeo nasus* [Boulenger](http://en.wikipedia.org/wiki/George_Albert_Boulenger), 1899 | AMNHI210-10  AMNHI431-11  DCF157-15 | BOLD | Lower and Middle Congo River Basin in Angola, Democratic Republic of the Congo and Central African Republic |
|  |  | *Labeo nunensis* Pellegrin, 1929 | AMNHI432-11  AMNHI434-11 | BOLD | Lower Guinea endemic, found in north-western Cameroon in Rivers: Noun, Djerem, Sanaga, Kelle, Mape, Assamba, Mbam, Mekay, Meng, Nchi, Nkoup, Mvi, Mevobo , tributary of Kim and northern Mifi |
|  |  | *Labeo parvus* [Boulenger](http://en.wikipedia.org/wiki/George_Albert_Boulenger), 1902 | JX074215.1  JX074209.1  JX074208.1 | GenBank | Widespread, from West Africa, including coastal Basins, Lake Chad and Niger River Systems to Congo River System |
|  |  | *Labeo rectipinnis* Tshibwabwa, 1997 | AMNHI161-10  AMNHI162-10 | BOLD | Democratic Republic of the Congo, Bas-Congo |
|  |  | *Labeo rosae* Steindachner, 1894 | HVDBF049-10  HVDBF064-10  HVDBF081-10 | ACDB, UJ | Limpopo River, Incomati and Usuto-Pongolo Basins. Also present in the Lufira River (Upper Congo River Basin) in Democratic Republic of the Congo |
|  |  | *Labeo senegalensis* [Valenciennes](http://en.wikipedia.org/wiki/Achille_Valenciennes), 1842 | BAFEN162-10  BAFEN167-10  BAFEN168-10  GBGC3899-07  GBGC3942-07 | BOLD | Senegal, Volta, Niger-Benue, Chad, Gambia and Culufi Rivers (West Africa |
|  |  | *Labeo simpsoni* Ricardo-Bertram, 1943 | AMNHI454-11  AMNHI456-11  AMNHI457-11  AMNHI458-11 | BOLD | Middle and Upper Congo River Basin in Cameroon, Central African Republic, Angola, Democratic Republic of the Congo and Zambia |
|  |  | *Labeo umbratus* (Smith, 1841) | HVDB299-10  HVDB300-10  HVDB301-10  HVDB302-10 | ACDB, UJ | Orange –Vaal System as well as the Gourits, Gamtoos, Sundays, Great Fish and Bushmans systems of the south and south-east Cape coastal regions. Has been translocated to the Keiskamma and Buffalo Systems in the Eastern Cape as well as the Olifants-Limpopo in Mpumalanga |
|  |  | *Labeo vulgaris* Heckel, 1847 | JX074220.1  JX074221.1  JX074222.1 | GenBank | Ethiopia |
|  |  | *Labeo weeksii* [Boulenger](http://en.wikipedia.org/wiki/George_Albert_Boulenger), 1909 | AMNHI217-10  AMNHI461-11  AMNHI464-11  AMNHI465-11  JX074184.1 | BOLD  GenBank | Widely distributed in the Congo River Basin; also in the Malagarasi Basin |
|  | *Labeobarbus* | *Labeobarbus caudovittatus* ([Boulenger](http://en.wikipedia.org/wiki/George_Albert_Boulenger), 1902) | GBHM418199.1  GBJX066759.1  GBKT192807.1  GBKT192808.1  GBKT192821.1 | GenBank | Middle and Upper Congo River System from Pool Malebo to Lake Mweru, in Democratic Republic of the Congo, Angola and Zambia. Also in Lake Tanganyika and associated Rivers such as the Ruzizi Basin |
|  |  | *Labeobarbus* *gananensis* Vinciguerra, 1895 | GBGCA6044-13  GBGCA6045-13  GBGCA6046-13 | BOLD | Juba and Awata rivers in Somalia. Reported from the Wabi Shebele Basin in Ethiopia |
|  |  | *Labeobarbus gorgorensis* (Bini, 1940) | JQ677095.1  GBGCA6068-13 | GenBank  BOLD | Lake Tana, Ethiopia |
|  |  | *Labeobarbus intermedius* (Rüppell, 1835) | GBGCA3113-13  GBGCA6047-13  GBGCA6048-13  GBGCA6049-13 | BOLD | Widely distributed throughout southern Ethiopia and into northern Kenya, certainly as far as Lake Baringo |
|  |  | *Labeobarbus kimberleyensis*  (Gilchrist & Thompson, 1913) | HVDB061-09  HVDBF711-12  HVDBF736-12  HVDBF739-12 | ACDB, UJ | Orange-Vaal River System |
|  | *Leptocypris* | *Leptocypris lujae* (Boulenger, 1909) | AMNHI007-09  AMNHI067-09  DCF565-15 | BOLD | Lower Congo River up to the Lualaba, including the Ubangi and Sankuru, in Democratic Republic of the Congo and Central African Republic |
|  |  | *Leptocypris modestus* Boulenger, 1900 | AMNHI031-09  AMNHI192-10  ANGBF2694-12  HM418158.1 | BOLD  GenBank | Lower Congo River up to the Upper Lualaba including the Ubangi, in Democratic Republic of the Congo and Central African Republic |
|  |  | *Leptocypris niloticus* (Joannis, 1835) | AMNHI215-10  ANGBF2686-12  HM224174.1  HM418179.1 | BOLD  GenBank | Rivers Nile, Omo, Niger, Bénoué, Volta. Senegal Rivers, and Lake Chad Basin |
|  |  | *Leptocypris weeksii* (Boulenger, 1899) | AMNHI032-09  AMNHI194-10  AMNHI211-10  HM418159.1 | BOLD  GenBank | Taia River in Sierre Leone and Little Scarcies River and Waanje River. Also reported from Rokel and Jong (Pampana) Rivers in Sierra Leone |
|  |  | *Leptocypris weynsii* (Boulenger, 1899) | AMNHI045-09  AMNHI067-09  AMNHI227-10  HM418187.1  HM418188.1 | BOLD  GenBank  GenBank | Middle Congo River up to the Upper Lualaba including the Ubangi and Kasai Drainages, in Democratic Republic of the Congo |
|  | *Luciobarbus* | *Luciobarbus biscarensis* (Boulenger, 1911) | FFMBH1430-14  FFMBH1433-14  FFMBH1434-14  FFMBH1435-14  FFMBH1436-14 | BOLD | Algeria, North Africa |
|  |  | *Luciobarbus callensis* (Valenciennes, 1842) | FFMBH1393-14  FFMBH1394-14  FFMBH1396-14  FFMBH1397-14  FFMBH1403-14 | BOLD | Tunisia, North Africa |
|  |  | *Luciobarbus issiensis* (Pellegrin, 1922) | FFMBH1504-14  FFMBH1505-14  FFMBH1506-14 | BOLD | Morroco, North Africa |
|  |  | *Luciobarbus ksibii* ([Boulenger](https://en.wikipedia.org/wiki/George_Albert_Boulenger), 1905) | FFMBH1342-14  FFMBH1463-14  FFMBH1467-14  FFMBH1493-14  FFMBH1494-14 | BOLD | Morroco, Igrounzar, North Africa |
|  |  | *Luciobarbus labiosa* Pellegrin, 1920 | FFMBH1345-14  FFMBH1349-14  FFMBH1454-14  FFMBH1455-14  FFMBH1499-14 | BOLD | Morroco, North Africa |
|  |  | *Luciobarbus lepineyi* ([Pellergrin](https://en.wikipedia.org/wiki/Jacques_Pellegrin), 1939) | FFMBH1351-14  FFMBH1352-14  FFMBH1361-14  FFMBH1362-14  FFMBH1363-14 | BOLD | Morroco, North Africa |
|  |  | *Luciobarbus leptopogon* (Schimper, 1834) | FFMBH1420-14  FFMBH1421-14 | BOLD | Algeria, North Africa |
|  |  | *Luciobarbus magniatlantis* (Pellegrin, 1919) | FFMBH1353-14  FFMBH1354-14  FFMBH1355-14  FFMBH1356-14  FFMBH2712-14 | BOLD | Morroco, North Africa |
|  |  | *Luciobarbus massaensis* (Pellegrin, 1922) | FFMBH1498-14  FFMBH1502-14  FFMBH1503-14 | BOLD | Morroco, North Africa |
|  |  | *Luciobarbus moulouyensis*  (Pellegrin, 1924) | FFMBH1456-14  FFMBH1457-14  FFMBH1458-14  FFMBH1460-14  FFMBH1461-14 | BOLD | Morroco, North Africa |
|  |  | *Luciobarbus nasus* (Guenther, 1874) | FFMBH1466-14  FFMBH1927-14  FFMBH2144-14  FFMBH2145-14 | BOLD | Morroco, North Africa |
|  |  | *Luciobarbus pallaryi* (Pellegrin, 1919) | FFMBH1338-14  FFMBH1339-14  FFMBH1364-14  FFMBH1365-14  FFMBH1470-14 | BOLD | Morroco, North Africa |
|  |  | *Luciobarbus setivimensis* (Valenciennes, 1842) | FFMBH1409-14  FFMBH1418-14  FFMBH1438-14  FFMBH1439-14  FFMBH1440-14 | BOLD | Morroco, North Africa |
|  | *Mesobola* | *Mesobola brevianalis* (Boulenger, 1908) | HVDB274-10  HVDB282-10  HVDB327-10  HVDB328-10  HM224176.1 | ACDB, UJ  GenBank | Cunene, Okavango, Upper Zambezi Systems and east coastal Rivers from the Limpopo to the Umfolozi in northern Natal. An isolated population is found in the Orange River below the Augrabies Falls. |
|  | *Opsaridium* | *Opsaridium boweni* (Fowler, 1930) | HM418193.1  HM418194.1  JX197000.1 | GenBank | Lulua River (Kasai Drainage, Middle Congo River Basin) in Democratic Republic of the Congo |
|  |  | *Opsaridium christyi* (Boulenger, 1920) | AMNHI062-09  AMNHI070-09 | BOLD | Republic of the Congo |
|  |  | *Opsaridium ubangiense* (Pellegrin, 1901) | DCF056-15  DCF057-15  DCF128-15  DCF129-15 | BOLD | Republic of the Congo, Cameroon |
|  | *Pseudobarbus* | *Barbus andrewi* now  *Pseudobarbus andrewi* [Barnard](http://en.wikipedia.org/wiki/Keppel_Harcourt_Barnard), 1937 | HVDBFT3-09  HVDBFT3-10  HVDBFT3-11  HVDBFT3-12  HVDBFT3-13 | ACDB, UJ | Berg and Breë Systems, south-western Cape Province, South Africa |
|  | *Raiamas* | *Raiamas batesii* (Boulenger, 1914 | HM418118.1  HM418119.1  HM418120.1  JX197002.1 | GenBank | Dja (Middle Congo River Basin), Sanaga and Nyong in Cameroon |
|  |  | *Raiamas buchholzi* (Peters, 1876) | HM418113.1  HM418114.1  HM418115.1 | GenBank | Coastal Basins of Cameroon to the Republic of Congo. Also present in the Rivers Ubangui and Sangha (Middle Congo River Basin) |
|  |  | *Raiamas kheeli* (Stiassny, Schelly & Schliewen, 2006 | HM418195.1  HM418196.1  HM418197.1 | GenBank | Lower Congo River in Democratic Republic of the Congo |
|  |  | *Raiamas salmolucius* (Nichols & Griscom, 1917) | JX197004.1  KT192831.1  KT192832.1  KT192904.1  KT192974.1 | GenBank | Congo River Basin and Lake Tanganyika |
|  |  | *Raiamas senegalensis* (Steindachner, 1870) | HM224215.1  HM224216.1 | GenBank | Basins of the Nile, Chad, Niger, Gambia, Senegal, Volta and coastal Basins of Sassandra, Bandama, Comoé, Tano, Pra, Ouémé, Ogun and Sanaga, which seems to represent its southern distributional limit . Also known from the Cross River in Cameroon |

**Sources**:

African centre for DNA barcoding, University of Johannesburg (ACDB, UJ)

Barcode of Life Database (BOLD; [www.boldsystems.org](http://www.boldsystems.org))

Froese R., Pauly D. 2016. FishBase. World Wide Web electronic publication. www.fishbase.org, versions (2006-2017)

GenBank/EBI ([www.ncbi.nlm.nih.gov/nuccore](http://www.ncbi.nlm.nih.gov/nuccore))

South Africa Institute of Aquatic Biodiversity (SAIAB)

Skelton P.H. 2001. A complete guide for fresh water fishes of southern Africa. Cape Town: Struik Publishers>
