# Supplementary material for: Re-evaluation of the discriminatory power of DNA barcoding on some specimens of African Cyprinidae (subfamilies Cyprininae and Danioninae)
Source: Zookeys. 2018 Mar 26;(746):105–21. doi: 10.3897/zookeys.746.13502 (PMC5906743; doi:10.3897/zookeys.746.13502)
Supplement: Supplementary material 3 — Table S3 [file zookeys-746-105-s003.docx]

| Species / characters | 1 | 2 | 3 | 4 | 5 | 6 |
| --- | --- | --- | --- | --- | --- | --- |
| *Barboides gracilis* | 1 | 1 | 0 | 0 | 2 | 0 |
| *Barbus* (*Pseudobarbus)andrewi* | 0 | 1 | 0 | 3 | 2 | 1 |
| *Chelaethiops bibie* | 2 | ? | 0 | 0 | 2 | 0 |
| *Chelaethiops congicus* | ? | 0 | ? | 0 | 2 | 0 |
| *Chelaethiops elongatus* | ? | ? | ? | 0 | 2 | 0 |
| *Clypeobarbus congicus* | ? | 0 | ? | 0 | 2 | 0 |
| *Clypeobarbus pleuropholis* | 1 | 1 | 0 | 0 | 2 | 0 |
| *Cyprinus carpio* | 0 | 1 | 2 | 2 | 2 | 2 |
| *Engraulicypris sardella* | ? | 0 | ? | 0 | 2 | 0 |
| *Enteromius aboinensis* | 1 | 1 | 0 | 0 | 2 | 0 |
| *Enteromius aspilus* | 1 | 1 | 0 | 0 | 2 | 0 |
| *Enteromius atromaculatus* | ? | 1 | ? | 0 | 2 | 0 |
| *Enteromius brazzai* | 1 | 1 | 0 | 0 | 2 | 0 |
| *Enteromius callipterus* | 1 | 1 | 0 | 0 | 2 | 0 |
| *Enteromius camptacanthus* | 1 | 1 | 0 | 0 | 2 | 0 |
| *Enteromius chiumbeensis* | ? | 1 | ? | 0 | 2 | 0 |
| *Enteromius fasciolatus* | 0 | 0 | 0 | 0 | 2 | 0 |
| *Enteromius holotaenia* | 0 | 1 | 0 | 0 | 2 | 0 |
| *Enteromius leonensis* | 1 | 0 | 0 | 0 | 2 | 0 |
| *Enteromius luluae* | ? | ? | ? | 0 | 2 | 0 |
| *Enteromius matthesi* | ? | ? | ? | 0 | 2 | 0 |
| *Enteromius mattozi* | 0 | 1 | 0 | 1 | 2 | 1 |
| *Enteromius miolepis* | 0 | 1 | 0 | 0 | 2 | 0 |
| *Enteromius paludinosus* | 0 | 1 | 0 | 0 | 2 | 0 |
| *Enteromius radiatus* | 0 | 0 | 0 | 0 | 2 | 0 |
| *Enteromius trimaculatus* | 0 | 0 | 0 | 0 | 2 | 0 |
| *Enteromius trinotatus* | ? | ? | ? | 0 | 2 | 0 |
| *Enteromius urostigma* | ? | ? | ? | 0 | 2 | 0 |
| *Garra congoensis* | ? | 0 | ? | 0 | 2 | 0 |
| *Garra dembeensis* | 1 | 0 | ? | 0 | 2 | 0 |
| Species / characters | 1 | 2 | 3 | 4 | 5 | 6 |
| *Garra ornate* | 1 | 0 | 1 | 0 | 2 | 0 |
| *Gyrinocheilus aymonieri* | ? | 0 | 0 | 1 | ? | 1 |
| *Labeo rectipinnis* | 1 | 0 | 0 | 0 | 1 | 0 |
| *Labeo altivelis* | 0 | 1 | 1 | 1 | 0 | 0 |
| *Labeo annectens* | ? | 0 | 0 | 2 | 0 | 0 |
| *Labeo ansorgii* | 0 | 1 | 0 | 1 | 0 | 0 |
| *Labeo barbatus* | ? | 1 | 0 | 2 | 1 | 0 |
| *Labeo batesii* | ? | ? | 0 | 1 | 1 | 0 |
| *Labeo capensis* | 0 | 1 | 0 | 1 | 0 | 0 |
| *Labeo coubie* | 1 | 0 | 2 | 3 | 1 | 0 |
| *Labeo cyclorhynchus* | 0 | 1 | 1 | 0 | 1 | 0 |
| *Labeo greenii* | ? | 1 | 0 | 1 | 1 | 0 |
| *Labeo lineatus* | ? | 0 | 0 | 3 | 0 | 0 |
| *Labeo longipinnis* | ? | 0 | 1 | 3 | ? | 0 |
| *Labeo lukulae* | ? | 0 | 1 | 1 | 1 | 0 |
| *Labeo lunatus* | 0 | 1 | 0 | 1 | 0 | 0 |
| *Labeo nasus* | 0 | 0 | 0 | 0 | 1 | 0 |
| *Labeo nunensis* | 1 | 0 | 1 | 1 | 1 | 0 |
| *Labeo parvus* | 1 | 0 | 1 | 0 | ? | 0 |
| *Labeo rosae* | 0 | 0 | 1 | 1 | ? | 0 |
| *Labeo senegalensis* | 1 | 1 | 1 | 3 | 0 | 0 |
| *Labeo simpsoni* | 0 | 0 | 0 | 1 | 1 | 0 |
| *Labeo umbratus* | 0 | 1 | 0 | 2 | 0 | 0 |
| *Labeo vulgaris* | ? | 0 | ? | 1 | ? | 0 |
| *Labeo weeksii* | ? | 0 | 1 | 1 | 0 | 0 |
| *Labeobarbus caudovittatus* | 1 | 1 | 1 | 2 | 2 | 2 |
| *Labebarbus gananensis* | ? | ? | ? | 0 | 3 | 0 |
| *Labeobarbus gorgorensis* | ? | ? | 0 | 0 | 3 | 0 |
| *Labeobarbus intermedius* | ? | 0 | ? | 2 | 3 | 2 |

| *Species/ characters* | 1 | 2 | 3 | 4 | 5 | 6 |
| --- | --- | --- | --- | --- | --- | --- |
| *Labeobarbus kimberleyensis*  *Leptocypris lujae* | 0  ? | 1  0 | 0  ? | 3  0 | 2  2 | 2  0 |
| *Leptocypris modestus* | ? | 1 | 1 | 1 | ? | 1 |
| *Leptocypris niloticus* | 1 | 0 | 1 | 0 | 2 | 0 |
| *Leptocypris weeksii* | ? | 0 | ? | 0 | 2 | 0 |
| *Leptocypris weynsii* | ? | 0 | ? | 0 | 2 | 0 |
| *Luciobarbus biscarensis* | 0 | 1 | ? | 1 | 2 | 1 |
| *Luciobarbus callensis* | ? | 1 | ? | 1 | 2 | 1 |
| *Luciobarbus issiensis* | ? | 1 | ? | 1 | 2 | 1 |
| *Luciobarbus ksibii* | ? | 1 | ? | 1 | 2 | 1 |
| *Luciobarbus labiosa* | ? | 1 | ? | 1 | 2 | 1 |
| *Luciobarbus lepineyi* | ? | 1 | ? | 1 | 2 | 1 |
| *Luciobarbus leptopogon* | ? | 1 | ? | 1 | 2 | 1 |
| *Luciobarbus magniatlantis* | ? | 1 | ? | 1 | 2 | 1 |
| *Luciobarbus massaensis* | ? | 1 | ? | 1 | 2 | 1 |
| *Luciobarbus moulouyensis* | ? | 1 | ? | 1 | 2 | 1 |
| *Luciobarbus nasus* | ? | 1 | ? | 1 | 2 | 1 |
| *Luciobarbus pallaryi* | 0 | 1 | ? | 3 | 2 | 1 |
| *Luciobarbus setivimensis* | ? | 1 | ? | 1 | 2 | 1 |
| *Mesobola brevianalis* | 2 | 0 | 0 | 0 | 2 | 0 |
| *Moxostoma breviceps* | ? | ? | ? | ? | ? | ? |
| *Opsaridium boweni* | ? | 0 | ? | 0 | 2 | 0 |
| *Opsaridium ubangiense* | 2 | 0 | 0 | 0 | 2 | 0 |
| *Pseudorasbora parva* | 1 | 0 | ? | 0 | 2 | 0 |
| *Raiamas batesii* | 0 | 0 | 0 | 2 | ? | 1 |
| *Raiamas buchholzi* | 2 | 0 | 0 | 0 | 2 | 0 |
| *Raiamas christyi* | ? | 0 | ? | 0 | ? | 0 |
| *Raiamas kheeli* | 2 | 0 | 0 | 0 | 2 | 0 |
| *Raiamas salmolucius* | ? | 0 | ? | 0 | 2 | 0 |
| *Raiamas senegalensis* | 2 | 0 | 0 | 0 | 2 | 0 |

**Summary of character descriptions**

1. Anal soft rays: 5-7 rays (0), 8-10 rays (1), 11-13 rays (2)

2. Barbs: absence (0), 1 pair (1), 2 pairs (2),

3. Dorsal soft rays: 7-10 (0), 11-14 (1), 15-18 (2)

4. Length: 1-20cm (0), 21-40cm (1), 41-60cm (2), 61-80cm and above (3)

5. Lip types: papillose (0), plicate (1), moderate (2), horny (3), thick (4)

6. Ploidy level: diploid (0), tetraploid (1), hexaploid (2)

7. ?: (Non available)

Outgroups: *Moxostoma brevicep*s*,* *Pseudobora parva* and *Gyrinocheilus aymonieri*

**Sources**:

African centre for DNA barcoding, University of Johannesburg (ACDB, UJ)

Barcode of Life Database (BOLD; [www.boldsystems.org](http://www.boldsystems.org))

Froese R., Pauly D. 2016. FishBase. World Wide Web electronic publication. www.fishbase.org, versions (2006-2016)

GenBank/EBI ([www.ncbi.nlm.nih.gov/nuccore](http://www.ncbi.nlm.nih.gov/nuccore))

South Africa Institute of Aquatic Biodiversity (SAIAB)

Skelton P.H. 2001. A complete guide for fresh water fishes of southern Africa. Cape Town: Struik Publishers

The IUCN Red List of Threatened Species. Version 2016-2. <[www.iucnredlist.org](http://www.iucnredlist.org/)>
